# Revisiting the Plasmodium falciparum druggable genome using predicted structures and data mining

**DOI:** 10.21203/rs.3.rs-5412515/v1

**Published:** 2024-11-26

**Authors:** Karla P. Godinez-Macias, Daisy Chen, J. Lincoln Wallis, Miles G. Siegel, Anna Adam, Selina Bopp, Krypton Carolino, Lauren B. Coulson, Greg Durst, Vandana Thathy, Lisl Esherick, Madeline A. Farringer, Erika L. Flannery, Barbara Forte, Tiqing Liu, Luma Godoy Magalhaes, Anil K. Gupta, Eva S. Istvan, Tiantian Jiang, Krittikorn Kumpornsin, Karen Lobb, Kyle McLean, Igor M. R. Moura, John Okombo, N. Connor Payne, Andrew Plater, Srinivasa P. S. Rao, Jair L. Siqueira-Neto, Bente A. Somsen, Robert L. Summers, Rumin Zhang, Michael K. Gilson, Francisco-Javier Gamo, Brice Campo, Beatriz Baragaña, James Duffy, Ian H. Gilbert, Amanda K. Lukens, Koen J. Dechering, Jacquin C. Niles, Case W. McNamara, Xiu Cheng, Lyn-Marie Birkholtz, Alfred W. Bronkhorst, David A. Fidock, Dyann F. Wirth, Daniel E. Goldberg, Marcus C.S. Lee, Elizabeth A. Winzeler

**Affiliations:** University of California, San Diego; University of California, San Diego; Panorama Global; Lgenia; MMV Medicines for Malaria Venture; Harvard T.H. Chan School of Public Health; University of California, San Diego; University of Cape Town; Lgenia; Columbia University Irving Medical Center; Massachusetts Institute of Technology; Harvard T.H. Chan School of Public Health; Novartis (United States); University of Dundee; University of California, San Diego; University of Dundee; Calibr-Skaggs Institute for Innovative Medicines; Washington University School of Medicine; University of California, San Diego; Calibr-Skaggs Institute for Innovative Medicines; Lgenia; Massachusetts Institute of Technology; Universidade de São Paulo; Columbia University Irving Medical Center; Harvard T.H. Chan School of Public Health; University of Dundee; Novartis (United States); University of California, San Diego; TropIQ Health Sciences; Harvard T.H. Chan School of Public Health; Global Health Drug Discovery Institute; University of California, San Diego; Global Health Medicines R&D; MMV Medicines for Malaria Venture; University of Dundee; MMV Medicines for Malaria Venture; University of Dundee; Harvard T.H. Chan School of Public Health; TropIQ Health Sciences; Massachusetts Institute of Technology; Calibr-Skaggs Institute for Innovative Medicines; Global Health Drug Discovery Institute; University of Pretoria; TropIQ Health Sciences; Columbia University Irving Medical Center; Harvard T.H. Chan School of Public Health; Washington University School of Medicine; Wellcome Centre for Anti-Infectives Research; University of California, San Diego

**Keywords:** druggable genome, Plasmodium blood stage targets, malaria data compendium

## Abstract

The identification of novel drug targets for the purpose of designing small molecule inhibitors is key component to modern drug discovery. In malaria parasites, discoveries of antimalarial targets have primarily occurred retroactively by investigating the mode of action of compounds found through phenotypic screens. Although this method has yielded many promising candidates, it is time- and resource-consuming and misses targets not captured by existing antimalarial compound libraries and phenotypic assay conditions. Leveraging recent advances in protein structure prediction and data mining, we systematically assessed the *Plasmodium falciparum* genome for proteins amenable to target-based drug discovery, identifying 867 candidate targets with evidence of small molecule binding and blood stage essentiality. Of these, 540 proteins showed strong essentiality evidence and lack inhibitors that have progressed to clinical trials. Expert review and rubric-based scoring of this subset based on additional criteria such as selectivity, structural information, and assay developability yielded 67 high priority candidates. This study also provides a genome-wide data resource and implements a generalizable framework for systematically evaluating and prioritizing novel pathogenic disease targets.

## Introduction

Over the past decade, phenotypic screening has gained popularity since large, diverse compound libraries can be tested for a desired therapeutic outcome in a high-throughput fashion without *a priori* knowledge of targets or mechanisms of action^1^. After triage, a subset of screening hits is typically subject to target identification, facilitating lead optimization and enabling the identification of new inhibitors through target-based drug discovery programs. In malaria parasites, this approach has successfully revealed new targets^1,2^, such as P-type cation translocating ATPase 4 (ATP4)^3^, acetyl-CoA synthetase (ACAS)^4^, translation elongation factor 2 (eEF2)^5^, Niemann-Pick Type C1-Related protein (NCR1)^6^, and several aminoacyl-tRNA synthetases^7–10^, which are urgently needed to develop drugs that differ from existing antimalarials in mechanism of action and resistance liability.

Compound-dependent target discovery, however, faces several limitations: available chemical matter to be tested is limited; primary screen design and hit prioritization restrict the space of targeted biology as only the most potent phenotypic hits are considered for follow-up characterization; and target identification is an arduous process, especially for novel targets lacking known ortholog inhibitors in other species. This process also tends to repeatedly identify targets such as *Pf*DHODH and *Pf*ATP4^1^. Alternatively, *in silico* approaches can systematically identify proteins amenable to target-based drug discovery^1,11,12^. For malaria parasites, key characteristics of a candidate target are its “druggability”, or ability to be modulated through high affinity binding of drug-like ligand(s), and its essentiality to parasite survival in the life cycle stage of interest. Although the druggable genome of the deadliest human malaria species, *Plasmodium falciparum*, has been explored through *in silico* methods^13–15^, previous studies have relied on homology to known targets and gene-drug interactions to predict druggability^14,16^. With the advent of artificial intelligence models for predicting protein structure, such as AlphaFold^17^ and ESMFold^18^, along with ligand binding prediction tools like AlphaFill^19^, we are now able to comprehensively assess the whole genome for essential proteins with evidence of small molecule binding. This approach may identify drug targets overlooked by compound-dependent discovery efforts.

Taking advantage of the collective expertise of the Malaria Drug Accelerator Consortium (MalDA)^2^, a collaborative partnership between academia and industry that aims to accelerate antimalarial drug discovery, we systematically identified and ranked a list of “druggable target” candidates from the entire *P. falciparum* genome that could progress in target-based drug discovery of novel therapeutics. The list was determined by identifying genes with evidence of protein binding to small molecules and evidence of essentiality in the parasite asexual blood stage (ABS); their viability as drug target candidates was further assessed based on common characteristics of known drug targets using available literature and data. As a result, we provide a list of promising blood stage antimalarial targets to pursue, as well as in-depth annotation resources that can facilitate future target validation and lead optimization efforts. In determining candidate targets through predicted ligand-target interactions from AlphaFill^19^, BindingDB^20^ and BRENDA^21^, we were able to uncovered small molecules that can be used as tool compounds for further studies or as starting points for structure activity relationship (SAR) design. The framework used in this study for synthesizing and evaluating information relevant to druggability may be applied to genome-wide *in silico* target discovery for other pathogenic organisms and diseases.

## Results

### Defining 1,660 P. falciparum genes with evidence of small molecule binding

**Figure 1:**
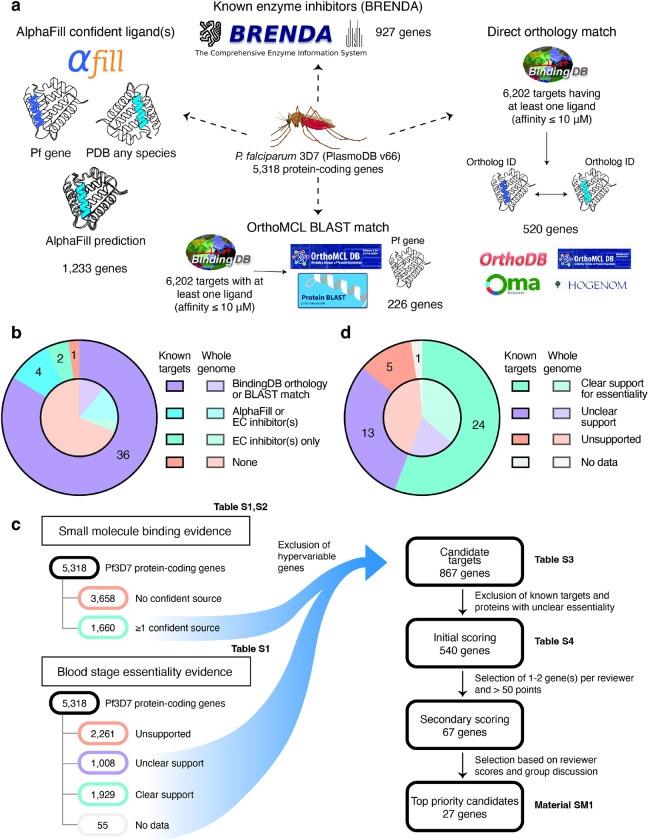
Identification of *P. falciparum* protein-coding genes that are potentially druggable (*n* = 1,660) and genes that have evidence of blood stage essentiality (*n* = 2,992). **a) Identification of potentially druggable genes.** Diagram illustrating the four methods used to identify genes with evidence of small molecule binding. Out of the 5,318 protein-coding 3D7 *falciparum* genes, 226 were found through a BLAST search using BindingDB 6,202 validated drug-targets, and 520 *falciparum* genes were found through orthology queries with OrthoMCL. A total of 1,233 genes had a confident AlphaFill hit transplant, and 927 *falciparum* genes were mapped through a validated EC number from BRENDA database. **b) Binding evidence for validated targets vs. all genes.** Distribution of binding evidence for 43 known targets^1^ (outer pie) compared to the distribution across all 5,275 *P. falciparum* 3D7 genes (inner pie). **c) Workflow for the identification of 867 candidate targets and subsequent prioritization.** Identification and filtering process to define the list of 867 *P. falciparum* candidate targets. The intersection of 1,660 genes with evidence of small molecule binding and 2,992 genes with essentiality support was used to determine the list of 867 candidates, after excluding hypervariable regions. Subsequent candidate prioritization resulted in 540 candidates that were subjected to initial scoring, 67 of which underwent a second round of scoring, and 27 top ranking targets discussed by a panel of experts that are likely to progress in the near future. **d) Essentiality classifications for validated targets vs. all genes.** Distribution of essentiality classifications for 43 known targets^1^ (outer pie) compared to the distribution of 5,275 3D7 genes (inner pie). Essentiality classifications are described in Methods.

Starting with 5,318 protein-coding genes in the *P. falciparum* 3D7 genome (PlasmoDB release 66), we identified a list of proteins that are “ligandable” and therefore potentially druggable. These proteins were arrived at by integrating data from predictions of ligands based on similarity to existing co-crystallized protein structures using AlphaFill^19^, orthology or sequence similarity to proteins with experimentally determined protein-ligand binding affinities in BindingDB^20^, and manually curated enzyme-inhibitor interactions in BRENDA^21^ (Fig. 1a, **Data S1–2**).

Of the 5,099 *P. falciparum* 3D7 genes with an associated AlphaFold protein model, 2,771 had at least one AlphaFill “hit”, i.e. sufficient local sequence homology to a protein in the PDB-REDO databank^19^ associated with ligand(s), referred as potential “transplants”. We restricted our attention to 1,233 proteins that had at least one confident AlphaFill transplant with global RMSD (root-mean-square-deviation, a measure of structural similarity) < 10 and local RMSD < 4, thresholds informed by empirical observation, while ignoring precipitants commonly used in protein crystallization and small ligands (< 10 atoms) which are unlikely to be drug-like (Methods). To broaden druggability evidence for *P. falciparum* enzymes and overcome the fact that many *P. falciparum* proteins are not orthologous to crystallized proteins, we incorporated information on inhibitors linked to EC (Enzyme Commission) number classes in the BRENDA database^21,22^. This yielded 321 additional proteins lacking confident AlphaFill predictions (**Data S1**). We further augmented our ligandable set by extracting 6,202 targets (UniProt IDs) from BindingDB^20^, a curated database of experimentally determined protein-ligand binding affinities, and performed phylogeny- and BLAST-based^23^ orthology queries for all 5,318 *P. falciparum* proteins (**Data S2**). Of these, 581 were orthologous to at least one of the 6,202 BindingDB targets based on OrthoMCL^24^, OMA^25^, HOGENOM^26^, or OrthoDB^27^ phylogenomic databases, or based on BLAST hits (E-value < 1) to the OrthoMCL full protein database.

Altogether, we found a total of 1,660 unique proteins with at least one source of small molecule-binding evidence. Many (*n =* 817) were identified by only one source (**Fig S1**), demonstrating the importance of considering multiple types of evidence to reduce false negatives. On the other hand, the set may include a few false positives. One possible example is the apical membrane antigen 1 (AMA1, PF3D7_1133400), an essential vaccine candidate lacking evidence of classical druggability that has been subjected to crystallography studies^28^. Authors used a peptide probe with the spin label MTSL, also known as (1-Oxyl-2,2,5,5-tetramethylpyrroline-3- methyl)-methanethiosulfonate, which was identified as an AlphaFill hit and happens to be of similar size to small molecule inhibitors^28^. Approaches more sophisticated than filtering based on molecular weight may be needed to remove these false positives, which highlight the need for expert review, as described below.

As a simple test, we examined a set of 43 known *P. falciparum* antimalarial targets that have some level of clinical, *in vivo*, or *in vitro* validation per a recent review by Siqueira-Neto et al.^1^, finding that, with the exception of NCR1 (PF3D7_0107500), all were supported by at least one source of binding evidence (Fig. 1b). Twenty-six of the 43 validated targets were well-known enzyme targets such as DHFR-TS (PF3D7_0417200) and DHODH (PF3D7_0603300) that had AlphaFill hit(s), ortholog(s) in BindingDB and known enzyme class inhibitors from BRENDA. In five cases (eEF2, elongation factor 2; CPSF3, cleavage and polyadenylation specificity factor subunit 3; FNT, formate-nitrite transporter FNT; PF3D7_1038900, a monoacylglycerol lipase-like esterase; and MQO, malate:quinone oxidoreductase), a single binding evidence source rescued the validated target.

### Defining 1,929 P. falciparum genes with evidence of blood stage essentiality

To assess which of the 5,318 *P. falciparum* protein-coding genes are required for asexual parasite growth, we incorporated essentiality data for *P. falciparum* and the rodent malaria species *P. berghei* (**Fig. S2**). We focused on the asexual blood stage (ABS) due to its role in the manifestation of clinical symptoms as well as completeness of available essentiality screens. In the Zhang et al. *falciparum* screen^29^, 3,271 proteins were labelled essential for *in vitro* ABS growth based on genome-wide transposon mutagenesis. Among 2,383 *falciparum* orthologs of *berghei* genes tested with gene disruption vectors in the PlasmoGEM dataset^30^, 1,145 were essential in ABS extrapolating from their *berghei* counterparts, while the RMgmDB dataset^31^ indicates change in phenotype upon gene modification for 1,319 of 1,609 *P. falciparum* genes whose *berghei* orthologs were tested^30,31^.

Reasoning that ambiguous essentiality data should not preclude proteins from consideration as targets, we created a categorization scheme for strength of essentiality evidence (**Fig. S3**). Categories were defined as “clear support”, “unclear support”, “unsupported”, or “no data” for essentiality in the asexual blood stage. For “clear support”, all available evidence sources must confidently label the protein as essential; if either Zhang et al. or PlasmoGEM confidently labels the protein non-essential, it was considered “unsupported”, while “unclear support” describes all other proteins with data from at least one source.

In total, 1,929 *P. falciparum* proteins were classified as having clear essentiality support, 1,008 with unclear support, 2,326 with support for non-essentiality, and 55 with no data (Fig. 1c). Surprisingly, based on this classification scheme five of the 43 validated targets from Siqueira-Neto et al. (MQO, PDEdelta, PNP, PF3D7_1038900, and PMX) were categorized under unclear support (Fig. 1d). In all but one case, either the Zhang et al. dataset or *P. berghei* datasets suggest the protein is essential while at least one source is contradictory. The exception was PDEδ (PF3D7_1470500, cGMP-specific 3’,4’-cyclic phosphodiesterase δ), which is not a blood stage target but rather the target of tadalafil in mature gametocyte stages where PDEδ regulates erythrocyte deformability^32^. While these results show that available data are sometimes inconsistent and can only partially inform *Plasmodium* blood stage gene essentiality, by incorporating extra layers of confirmation with *in vitro* and *in vivo* evidence where available, we increased our confidence that proteins in the “clear support” category are essential and thus more likely to be valuable drug targets.

### 867 P. falciparum proteins have evidence of binding and blood stage essentiality

**Figure 2:**
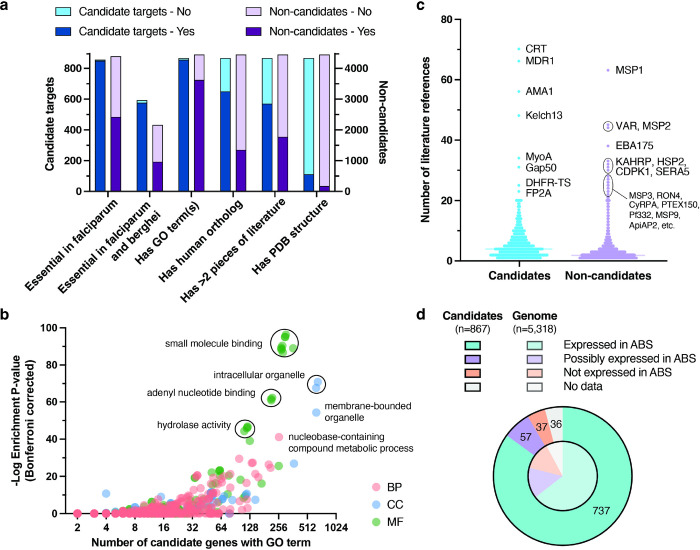
Characteristics of 867 candidate targets compared to 4,451 non-candidate genes. **a) Characteristics of candidate targets vs. non-candidates.** Comparison of characteristics between the 867 candidates (blue) and 4,451 non-candidates (purple). Numbers of candidate targets vs non-candidates labelled essential only in the *P. falciparum* essentiality screen and not the *P. berghei* datasets (850 vs. 2,421); labelled essential in both *P. falciparum and berghei* datasets (577 vs. 967); having at least one GO term (857 vs. 3,624); having human ortholog(s) (650 vs 1,356); having >2 associated literature references (570 vs 1,775); or having PDB structures (112 vs 174) are shown. **b) Gene ontology (GO) term enrichment analysis for the 867 candidates.** GO term enrichment analysis for the 867 candidate targets compared to all 5,318 protein coding genes in the *P. falciparum* 3D7 genome using GOATOOLS^85^. Terms with ontology tree depth > 2 (*n =* 939) are displayed based on number of candidates having the GO term (X-axis) versus −log_10_ Bonferroni corrected enrichment *P* value, with a maximum uncorrected *P*-value of 0.05 (Y-axis, Fisher’s exact test). Points corresponding to GO terms are colored by ontology type: red for biological process (BP), blue for cellular component (CC), and green for molecular function (MF). Highly enriched terms or groups of terms are labelled with shared descriptors. **c) Scientific literature references for candidates vs. non-candidates.** Distributions of number of unique scientific publications associated with the 867 candidate targets (blue) vs. 4,451 non-candidate genes (purple). Median lines are shown for both groups, and the most highly referenced genes are labelled. **d) Gene expression in the asexual blood stage (ABS) compared to all protein-coding genes.** Distribution of classifications for evidence of gene expression in the asexual blood stage (ring, trophozoite, or schizont) according to Le Roch et al. data^41^. Candidate targets with clear evidence of ABS expression (737, teal), unclear evidence (57, purple), no expression (37, orange) and no data (36, grey) are shown in the outer pie, in contrast to the distribution among all *P. falciparum* protein-coding genes in the inner pie.

To define an initial list of ligandable and essential candidate targets, we took the intersection of the set of 1,660 proteins with small molecule binding evidence and the set of 2,992 proteins not categorized as “unsupported” in terms of blood stage essentiality (Fig. 1c). This yielded 867 candidate targets after filtering out 19 genes in hypervariable non-core regions (**Data S3**). Non-core genes, encompassing *var*, *rifin* and *stevor* multigene families and other genes in highly recombinogenic subtelomeres^33,34^, were not considered as their variability and redundancy make them poor targets despite a few cases where they were deemed essential (e.g. PF3D7_0101600, a rifin, has a mutagenesis index score of 0.199^29^). The 867 candidate targets were distributed throughout the genome with no apparent propensity for specific chromosomes (**Fig. S4**). Most (*n =* 651) of the 867 candidate targets were supported by binding evidence from AlphaFill, 336 were orthologs of or had BLAST matches to validated targets in BindingDB, and 457 were supported by BRENDA enzymatic data. Among 857 candidate targets present in the Zhang et al. *P. falciparum* dataset, 850 were labelled as essential, in contrast to 2,421 of 4,396 non-candidate proteins (Fig. 2a).

Attractively, 577 of the 867 candidate targets were found to be confidently essential in both *falciparum* parasites and the PlasmoGEM (*n* = 452) or RMgmDB (*n* = 162) *berghei* datasets (Fig. 2a). This suggests their potential as therapeutic targets for more than one *Plasmodium* species, which is important given that most antimalarial drugs will need to act against *P. vivax* and *P. malariae*. Among these 577 candidates, we observed known targets (*n* = 7) with validated clinical inhibitors, including eEF2 and PI4K (phosphatidylinositol 4-kinase), as well as attractive yet clinically unexplored targets such as BDP1 (PF3D7_1033700, bromodomain protein 1), which has been subjected to phenotypic analysis^35^ and has an apo crystal structure^36^. On the other hand, only a small number of candidate proteins (*n =* 14) appeared to be confidently essential in *falciparum* but not *berghei*, including two acyl-CoA synthetases (PF3D7_0301000, PF3D7_0525100) and two serine/threonine FIKK kinases (PF3D7_0301200, PF3D7_0902400).

### Building an annotation resource using scientific evidence to prioritize candidate targets

To more thoroughly characterize the 867 candidate targets, we compiled additional annotations for all *P. falciparum* protein-coding genes (**Fig. S5**). We included information on genomic features and genetic variation (PlasmoDB^37^, NCBI^38^ and MalariaGEN^39^), protein features and structures, expression across malaria parasite life cycle stages (Malaria Cell Atlas^40^ and Le Roch et al.^41^), literature references (NCBI, PubMed), and similarity to human orthologs (Fig. 2a, **Data S3**) (see Methods for more details). We reasoned that in addition to druggability evidence, these annotations would allow us to prioritize proteins that merit further structural/functional characterization and target-based screens. The compiled data are displayed for each gene via a web resource, available online at http://pftargetbrowser.org, and summarized in **Data S3**.

Across the *P. falciparum* genome, only 286 of 5,318 proteins have an experimentally determined structure in the Protein Data Bank (PDB)^42^; of these, 112 were in our list of 867 candidate targets, reflecting substantial prior characterization of many of the candidate targets and highlighting those amenable to structure-based drug design (Fig. 2a). Examples of candidate crystal structures include ferredoxin-NADP reductase (FNR)^43^ and aspartate carbamoyltransferase (ATCase) in complex with a recently discovered small molecule allosteric inhibitor^44^. We also observed that 2,006 protein-coding genes have human orthologs based on OrthoMCL; to estimate structural similarity of *P. falciparum* proteins to their human counterparts, which plays a key role in selectivity and thus therapeutic side-effects, we ran pairwise TM-align^45^ comparisons of their AlphaFold models. This allowed us to identify the most similar human ortholog for 1,972 *P. falciparum* proteins (AlphaFold structures were not available in 34 cases), showing an average sequence identity of 33% for local alignments that were, on average, 244 amino acids long (**Fig. S6**). A human ortholog was not reported for 217 candidates, which may include promising targets involved in parasite-specific essential biology.

To characterize the biological functions of proteins in the candidate list, we performed Gene Ontology (GO) term enrichment analysis (Fig. 2b). Across the 867 candidates, of which 857 had at least one associated GO term, the nine most highly enriched terms with ontology tree depth > 2 were related to small molecule binding, in particular nucleotide binding (*n* = 299, *P* = 8.2 × 10^−96^, Bonferroni corrected). Following behind, the cellular component term “intracellular organelle” was also highly enriched (*n =* 659, *P* = 1.2 × 10^−71^). Closer inspection showed that these 659 candidates have greater proportions of genes associated with nucleus (*n =* 371, *P* = 1.3 × 10^−27^), endoplasmic reticulum (*n* = 54, *P* = 9.5 × 10^−7^), food vacuole (*n* = 50, *P* = 6.2 × 10^−12^), and other intracellular organelles compared to all protein-coding genes. Other overrepresented GO terms among candidate targets include ATP binding (*n* = 213, *P* = 2.3 × 10^−62^), pyrophosphatase activity (*n* = 119, *P* = 1.4 × 10^−46^), and more. These results suggest that the candidate list successfully differentiates proteins that are essential and ligandable from those that are not on the basis of cellular function and localization.

To assess availability of prior evidence permitting researchers to make informed hypotheses and, ultimately, more efficacious therapeutics, we queried literature repositories using PlasmoDB and Entrez gene identifiers (Fig. 2c). Through this approach, we were able to rescue evidence before *Plasmodium* gene nomenclature was standardized; for example, four additional references for *pfhsp101* (PF3D7_1116800) were recovered. Overall, we found literature references for 4,956 genes, with medians of four references per gene among candidate targets and two references per gene among non-candidates (Fig. 2c). Unsurprisingly, well-studied genes such as the multidrug resistance genes *pfcrt* (70 references) and *pfmdr1* (66 references), and vaccine targets such as *pfmsp1* (63 references) and *pfama1* (56 references), had the most references.

Finally, to identify candidates that could be targeted at multiple stages, we examined evidence of gene expression across the parasite life cycle using the Le Roch et al. microarray dataset^41^, which remains useful because it includes a probability of detection above background. As expected, 85% (*n =* 737) of the 867 candidate targets were strongly supported by expression in at least one ABS substage, in contrast to 64.5% of all *P. falciparum* genes (Fig. 2d). Of these 737 candidates, 577 also showed strong evidence of expression in the sexual (gametocyte) or mosquito (sporozoite) stages. The remaining 130 candidates were either not measured (*n =* 36), have unclear expression (*n =* 57), or were clearly not expressed across ABS substages in the microarray dataset (*n =* 37). Around half of these 37 candidate genes also appeared to be minimally expressed according to ABS scRNA-seq data from the Malaria Cell Atlas study^40^, while the other half either contradicted Malaria Cell Atlas expression levels or had dubious evidence of essentiality. In the latter case, many were small with protein sizes on the order of 100 amino acids, which have a lower probability of being detected with both RNA-seq and with tiling microarrays. It is possible that some proteins such as RPUSP (RNA pseudouridylate synthase), CYC4 (cyclin), and YTH1 (YTH domain-containing protein 1) are essential despite being expressed at low levels, which could be an advantageous property as an antimalarial target^46^.

While this work focuses on prioritization of blood stage targets for which essentiality and expression data is the most complete, we observed 196 *P. falciparum* orthologs of *P. berghei* genes showing evidence of essentiality in the liver stage^47^ but not in the ABS stage. Of these orthologs, 104 have binding evidence, suggesting their potential as liver stage-specific prophylactic antimalarial targets.

### Scoring 540 novel candidate targets with strong evidence of essentiality

We next sought to narrow down the candidate targets to those that have strong evidence of essentiality and are relatively novel (lack of prior characterization, especially as an antimalarial target) for further evaluation and prioritization. Starting with 587 candidate targets classified as having “clear support” for blood stage essentiality, we filtered out well-known antimalarial targets, such as DHODH and DHFR-TS, validated targets, and target classes currently being pursued by MalDA or other groups, such as aminoacyl tRNA synthetases^2^. This resulted in a list of 540 understudied (novel) candidate targets with at least one piece of binding evidence that are more likely to disrupt parasite growth and survival upon perturbation (Fig. 1c).

**Figure 3:**
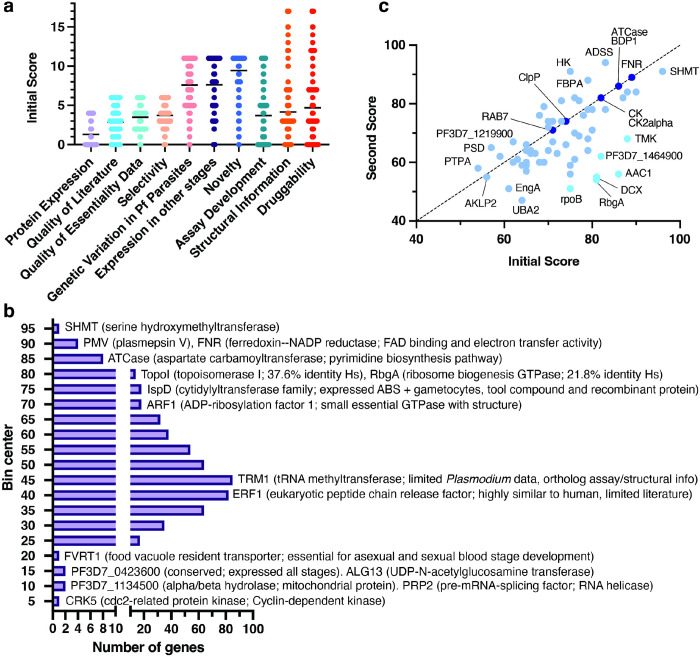
Rubric-based scoring of 540 candidate targets with strong evidence of essentiality. **a) Gene scores across rubric categories.** Plot showing first score distributions for 540 candidate targets with strong evidence of blood stage essentiality across the ten categories of the scoring rubric (Methods). Average value per category is shown for each category. **b) Frequency distribution of total first score.** Histogram showing the frequency (X-axis) of first total score (Y-axis) for the 540 scored candidates. Bin center was determined and plotted with Prism v.9.5. Examples of candidates falling in select total score bins are shown, including gene product description and notable characteristics when applicable. **c) Comparison of first and second scores for the top 67 scored candidates subject to secondary review.** Identity line is marked by a dashed black line. Dark blue circles denote equal score in both rounds; light blue circles represent score differences of 1–19 points; and cyan circles represent a score difference of at least 20 points.

Taking advantage of the data compendium, we created a rubric (see Methods) to manually score each of the 540 candidate targets based on their potential for progressing into antimalarial target-based drug discovery (Fig. 3a, **Data S4**). The rubric was designed to consider quantity and quality of compiled evidence, readiness of functional or binding assay development, and novelty across scientific literature. Briefly, ten categories summing up to a maximum of 100 points were scored per target, aiming to highlight novel proteins with strong support across all categories; points were deducted for weak, missing, or contradictory evidence. When suitable, expert reviewers suggested advancing or deprioritizing a candidate target. For example, reviewers deprioritized CK2α and FKBP35 due to concerns about lack of effect on asexual growth from conditional knockout studies^48,49^.

Under this proposed rubric, scores for the 540 novel candidate targets ranged from 6 to 96 points, with an average score of 48.64 (**Data S4**). Candidates generally received high scores when prior characterization had been done, while lower scores (≤45) were assigned in the absence of recombinant protein expression, biochemical assays, and structural or druggability information. Among 255 low-scoring candidate targets, we observed subunits of protein complexes, challenging enzyme classes like GTPases, and unsuccessful pre-clinical targets in any organism. For example, RRP45 (PF3D7_1364500), an RNA exosome complex component, scored 36 points due to lack of successful recombinant protein production, lack of a biochemical assay and limited protein structure and tool compound information. Nevertheless, targets with limited prior work also scored high in novelty according to our metric (**Data S4**).

Our scoring also revealed attractive high-scoring candidates. One example is TopoI (PF3D7_0510500, topoisomerase I; 80 points), involved in DNA replication, transcription, and repair (Fig. 3b). A bacterial TopoI inhibitor^50^ is known, suggesting that the *Plasmodium* enzyme could be selectively targeted. Although our attention was drawn to high-scoring genes, those with lower scores still have potential as drug targets. Such candidates, including the NAD kinase PF3D7_0913300 or proteins that lack human orthologs but are conserved within natural parasite populations like PF3D7_1356600 (predicted regulator of chromosome condensation) and PF3D7_1446800 (heme detoxification protein), will require substantial additional research to confirm their viability as antimalarial targets.

### Secondary scoring of 67 high-ranking candidate targets

Although candidate targets were scored according to a predefined rubric, scores were manually determined and could thus vary among different reviewers. For example, a reviewer may give a higher score if there is an enzymatic assay specifically for the enzyme under review, whereas another reviewer could give the same score if an enzymatic assay is available for the enzyme class. Therefore, to increase confidence in the scores, we conducted a second round of scoring for 67 high-ranking candidates (Fig. 3c, **Data S4**). These 67 candidates were selected by aggregating up to two of the highest scored proteins recommended by each of the initial reviewers with a minimum first score of 50.

Secondary scoring for the 67 candidates averaged 69.22 points, slightly lower than the first round (73.55 points). Six candidates showed a difference of more than 20 points (Fig. 3c). One example, ribosome biogenesis GTPase A (RbgA), decreased in score from 81 to 54, as the second reviewer placed greater emphasis on the lack of a tool compound and the fact that recombinant protein was only expressed in bacteria. On the other hand, seven genes received the same score from independent reviewers, including ATCase and FNR (Fig. 3c), supporting the rubric’s utility in prioritizing candidate targets.

### In-depth consideration of 27 prioritized candidates reveals targets poised for drug discovery

From the 67 high-ranking candidate targets with secondary scores, 27 were selected for in-depth consideration by a panel of MalDA experts by once again aggregating the top 1–2 candidates recommended by each secondary reviewer. Assessments of target-based drug discovery resources, follow-up strategies, and enablement challenges for the 27 prioritized targets are summarized in **SM A1**. Among these targets, we found several caseinolytic protease ATPases (ClpQ, ClpS, ClpY, ClpP, ClpB1), which play important roles in protein homeostasis, and enzymes in the methylerythritol phosphate isoprenoid biosynthesis pathway (IspD, IspE, IspF) that were independently highlighted by different reviewers; both groups of proteins are apicoplast-targeted and lack human homologs, favoring inhibitor selectivity.

**Figure 4:**
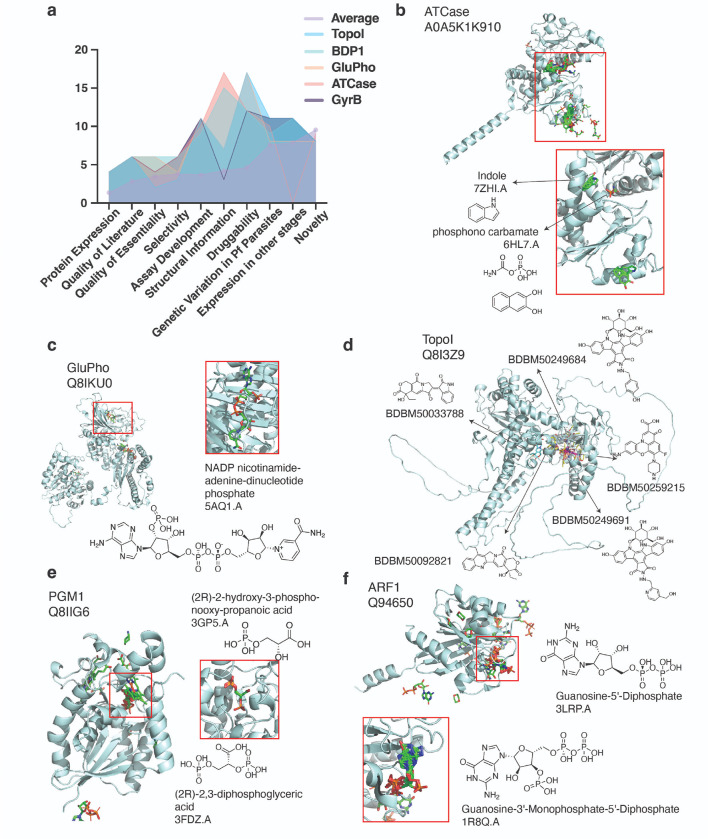
Selection of five high-scoring targets and examples of two low-scoring but promising candidates. **a) Scoring distribution across categories for top five candidate targets.** Individual scores for the top five candidate targets across rubric categories, compared with the average scores across all 540 scored candidates. The average score is highlighted by light purple circles, and top five candidates are shown in blue (TopoI), green (BDP1), orange (GluPho), salmon (ATCase) and dark purple (GyrB). **b-c) AlphaFill models for advanced candidate targets.** Predicted AlphaFill models for PfATCase (**b**) and PfGluPho (**c**) are shown. Red rectangles highlight the region where transplant hits were found, with a zoomed-in inset of hit transplant structure having highest percentage of identity. **d) TopoI model.** TopoI (PF3D7_0510500) was constructed using UniProt ID Q8I3Z9 and ligand hits (**Data S2**). For simplicity, five ligands (BDBM-50249684, -50033788, -50259215, -50249691, and -50092821) associated to the UniProt ID were randomly selected from BindingDB hits. Ligands were docked onto the model using openbabel 3.1.1^86^ and smina 2020.12.10^87^. The model was visualized using PyMol version 2.5.5^88^. **e-f) AlphaFill models for understudied but promising candidate targets.** Predicted AlphaFill models for PfPGM1 (**e**) and PfARF1 (**f**) candidate targets. Red rectangles highlight the region of some transplant hits, and a zoomed-in inset including hit transplant structure with highest percentage of identity is shown.

This exercise also highlighted five attractive targets: ATCase, TopoI, GyrB (DNA gyrase subunit B), GluPho, and BDP1 (Fig. 4a, **Data S4**). These five targets show minimal concerns for their progression into drug discovery efforts according to evaluated categories, with all but BDP1 having previously demonstrated small molecule inhibitors^51–54^. Below, we describe ATCase, GluPho, and TopoI, targets closer to lead optimization studies.

ATCase (aspartate transcarbamoylase; Fig. 4b) is a 43.3 kDa protein catalyzing the second step in *Plasmodium*’s de novo pyrimidine synthesis pathway, forming a homo-trimer with three active sites^54^. This pathway is clinically essential since parasites lack a pyrimidine-import pathway, reflected by inhibitors targeting *Plasmodium* DHODH, a downstream enzyme^54^. A truncated version of ATC has been successfully cloned and expressed, and PfATCase has been crystallized as an apo structure and with a bound allosteric inhibitor, with nearly 40% homology to the catalytic subunit of *E. coli* ATC^44,55,56^. This enzyme can be measured with phosphate- and carbamoyl aspartate-based assays^56^ and has good selectivity, as PALA analogs, T-state inhibitors, and allosteric inhibitors are effective against human ATCase, but not PfATCase^54^. Torin2, an ATP-competitive inhibitor, exhibited micromolar potency (PfATCase IC_50_ = 67.7μM)^57^, while the ligand 2,3- naphthalenediol has medium potency (IC_50_ = 5.5 μM)^54^ in addition to non-druglike features including high aromaticity. Additional SAR or evaluation of new libraries are needed to identify more suitable starting points for drug discovery and inhibitors with tight binding potential.

GluPho (glucose-6-phosphate dehydrogenase-6-phosphogluconolactonase) is another attractive validated target (Fig. 4c). This bifunctional enzyme catalyzes the first two steps in the pentose phosphate pathway which serves as the major source of NADPH in *Plasmodium*, critical for maintaining parasite redox equilibrium in infected red blood cells^51,58^. Several selective GluPho inhibitors have been identified through target-based screens for *P. falciparum* (e.g. ML276, IC_50_ = 0.89 uM^59^; SBI–0797750, IC_50_ = 0.007 uM^60^; ML304, IC50 = 0.19 uM^61^) as well as other organisms such as *Saccharomyces cerevisiae* (e.g. the catechin gallate compound CHEMBL408233 IC_50_ = 21.76 uM^62^). As current ligands have liabilities, further work on known series and high-throughput screening for PfGluPho inhibitors is warranted.

TopoI (topoisomerase I) (Fig. 4d), a highly conserved and essential nuclear enzyme, is the only type IB topoisomerase among seven *P. falciparum* topoisomerases^53^. Topoisomerases are well-established targets of anticancer and antibacterial drugs, which act as cellular poisons by selectively trapping the enzyme-DNA cleavage complex^63^. Camptothecin, a classic topoisomerase inhibitor, is potent against erythrocytic parasites^64^, and TopoI shows the highest endogenous activity in schizonts based on functional assays measuring relaxation of supercoiled plasmid DNA, suggesting its role in DNA replication during schizogony^65^. Recombinant expression systems, functional assays, and tool compounds, including some with whole cell anti-parasite activity, are available for PfTopoI, although selectivity remains a challenge^53,64–66^.

### Secondary reviews suggest 29 understudied candidate targets meriting further characterization

In addition to assessing proteins that were previously explored as antimicrobial targets, our scores inform the feasibility of understudied proteins progressing as novel antimalarial targets. Of the 67 candidate targets with secondary reviews, we find 29 receiving the maximum novelty score of 11 points (**Data S4**). Although some characterization is available for these candidates, substantial work is needed to confirm their viability as antimalarial targets. PGM1 (Fig. 4e) and ARF1 (Fig. 4f), highly novel candidate targets with average scores of 73.5 and 71, respectively, are discussed below.

PGM1 (phosphoglycerate mutase) is involved in glycolysis and gluconeogenesis^67^. It is essential in *falciparum* and *berghei* parasites^29,31^, expressed in multiple stages^41^, and although it has significant similarity to the human enzyme (56.8%, TMalign score = 0.9769), differences in protein quaternary structure of tetramer (*P. falciparum*) versus dimer (human) suggest potential for selectivity. Furthermore, conditional knockdown of PfPGM1 resulted in growth arrest, consistent with the predicted essentiality of the target^67^. Although inhibitors have not been found, several starting points for validation studies (e.g., selectivity and druggability) and tool compound SAR development against PfPGM1 are available.

ARF1 (ADP-ribosylation factor 1) is a GTPase involved in secretory protein trafficking in eukaryotic cells by initiating vesicle formation at the Golgi apparatus. Our analysis indicates that this enzyme is essential in *falciparum* and *berghei* parasites, expressed in sporozoite, gametocyte, and asexual blood stages^40,41^, and has multiple confident AlphaFill transplant hits. Studies have shown that this enzyme plays an important role in cancer metastasis; substantial work on human ARF1 has identified diverse inhibitors ranging from the octahydronaphthalene derivative AMF-26^68^ to the triterpenoid natural product demethylzeylasteral^69–71^, providing clues on a potential therapeutic strategy for malaria parasites. Although ARF1 has several favorable characteristics, i.e. crystal structure and inhibitors in *Plasmodium* and cancer cells, computational prediction and experimental validation are needed to identify effective and potent *Plasmodium* inhibitors since a general druggability challenge with small GTPases is the displacement of GTP binding.

## Discussion

In this study, we present a systematic data compendium of the *Plasmodium* genome focused on druggability potential as well as an updated set of potential targets that can readily progress into drug discovery programs. To assess evidence for druggability, we leveraged the AlphaFill database of predicted ligand “transplants” based on homology of AlphaFold structures to all structures in the PDB-REDO databank, setting the basis for SAR studies. One concern with this approach is that due to lax criteria for binding and essentiality evidence, the list of 867 “potentially druggable” candidate targets is likely to contain false positives. Many AlphaFill-predicted ligand hits were generic molecules such as ATP which may not translate to drug-like inhibitors; more sophisticated filtering of AlphaFill hits based on chemical properties may improve the positive predictive value of this strategy. In addition, far fewer crystal structures exist for *Plasmodium* and apicomplexan parasites compared to other organisms, such as mouse or human; as a result, predicted *P. falciparum* transplant hits found with distant orthologs may not be suitable for malaria parasites, reflected in low “druggability” scores during expert evaluation.

To minimize these issues, at the cost of deprioritizing completely novel *Plasmodium*-specific candidate targets, we focused on proteins with additional sources of binding evidence such as validated inhibitors in other species. Further validation of predicted ligand “transplants” with putative *P. falciparum* protein targets will be needed, which may take several months of SAR to improve affinity strength and inhibitory potency. On the other hand, some proteins with entirely novel modes of binding may be absent from the candidate set as they lack a clear binding pocket or predicted ligand, but are in fact ligandable via a cryptic pocket, i.e. one absent in crystal structures but apparent upon binding of the right ligand. Such cryptic pockets may enable targets in protein classes historically considered undruggable, as in the case of the mutant K-Ras inhibitors^72,73^. Molecular dynamics and/or deep learning approaches to binding pocket prediction may rescue potential false negatives^74–79^.

Another limitation is that manual scoring was only performed on 540 candidate targets with strong evidence of ABS essentiality, while targets with ambiguous or conflicting phenotypes based on gene disruption studies were overlooked. In some cases, an intermediate relative growth rate labelled “slow” by PlasmoGEM prevented the classification of genes as clearly essential, such as for the known antimalarial target ATP4. While the *P. berghei* essentiality datasets served to validate results from the *P. falciparum* mutagenesis screen, which are less reliable for genes that are small or have low TTAA density, essential genes in *P. falciparum* may not be essential in other species. Additional species-specific essentiality datasets can further provide insight into the landscape of essential *falciparum* genes across its life cycle.

The target evaluation rubric in this study favored proteins with substantial prior characterization and assay development, facilitating immediate follow-up validation and screening work. Due to this focus on “low-hanging fruit”, genes fulfilling alternative criteria, such as hitherto unexplored target classes or *Plasmodium*-specific genes of unknown function, were not highlighted by our ranking. Nevertheless, essential genes with confidently predicted binding hit(s) provide an initial hint that may result in novel target classes, though substantial follow-up efforts are needed since they lack key target fulfillment data.

To date, clinically effective antimalarials with known mechanisms have been limited to drugs targeting known druggable proteins, i.e. those with well-defined, specific hydrophobic pockets that bind small molecule ligands. Our study therefore focused on systematically identifying classically druggable proteins, which are more likely to yield small molecule inhibitors that tend to have favorable oral bioavailability, stability, affordability, etc. In addition to cryptic pockets, many new approaches to targeting “undruggable” proteins have emerged, such as allosteric inhibitors modulating protein-protein interactions, RNA therapeutics utilizing antisense oligonucleotides or RNAi, or PROTAC (proteolysis-targeting chimera) technology^80^. Thus, it is possible that essential *P. falciparum* genes that lacked small molecule binding evidence in our analysis could be targeted through alternative methods.

We believe the list of gene candidates proposed in this work can serve as a starting point for future phenotypic validation and small molecule optimization efforts. As new information about protein structure and gene function is constantly being generated, an automated extraction and integration of data will be the next step towards a dynamic resource for prioritizing novel antimalarial targets. We also believe that the target evaluation approach described can be applied to other disease-causing organisms, as exemplified by a similar exercise to rank targets in *Mycobacterium tuberculosis*^12^. For *P. falciparum* malaria, our data compendium may assist in prioritizing genes for other use cases, such as vaccine development. Overall, we believe this project and the web-based data portal will serve as a valuable resource for the malaria community and assist in directing resources and effort towards future high-quality drug targets.

## Methods

### Data acquisition

List of genes and genomic features (GFF) for *Plasmodium falciparum* 3D7 genome (PlasmoDB release 66) was downloaded and protein coding genes were extracted along with their gene annotations and genomic location. Additional genomic annotations were obtained by querying PlasmoDB to extract UniProt and Entrez ID(s), ortholog group (OrthoMCL), protein features (CDS and protein length, molecular weight, isoelectric point), domain annotations (InterPro, PFam, Superfamily), number of transmembrane (TM) domains, and enzyme commission (EC) numbers. Gene function (Gene Ontology; components, functions and processes) was extracted by either PlasmoDB or by querying the InterPro ID under InterPro2GO mapping tool from EMBL-EBI services. Gene essentiality data was obtained for *P. falciparum*^29^ and *P. berghei*^30,31^ parasites that were mapped to their *falciparum* ortholog using OrthoMCL orthology group IDs. Protein Data Bank (PDB) IDs of crystal structures were obtained by searching either gene symbols, UniProt IDs associated with each gene, or by typing “*Plasmodium*” in the PDB website search box. A report with gene identifier, organism, accession number, method for structure determination and publication information was extracted for the search hits.

### Mapping genes to associated literature publications

A download from the NCBI FTP site was performed for gene2pubmed.gz (version 2024–02-21) containing taxonomy ID, gene ID (Entrez) and PubMed ID. Gene IDs were mapped to the *P. falciparum* 3D7 annotation set, and PMIDs matching the criteria were extracted. To include literature references associated with gene symbols, we queried each gene symbol in PubMed using the Eutils^81^ efetch function from NCBI; additional information for each publication was obtained pragmatically using the same tool, retrieving title, authors and DOI (digital object identifier). Literature references from gene nomenclature extraction were manually reviewed and filtered for unrelated records (e.g., same name but different meaning across organisms/disease).

### Determining candidate proteins with evidence of small molecule binding

BindingDB^20^ (version 2024–01-01) was queried to extract a list of 6,202 unique UniProt IDs with at least one ligand having a measured affinity of at least 10 μM. Ligand SMILES were extracted for target hits. The proteins in this list were queried against a custom OrthoMCL^24^ (v.6.19) database with BLAST v2.15 blastp function^23,82^. Orthology of *P. falciparum* 3D7 proteins to any of the 6,202 BindingDB proteins was determined based on presence in the same ortholog group according to OrthoMCL, HOGENOM^26^, OMA^25^ and OrthoDB^27^ phylogenomic databases, using the UniProt ID mapping tool (accessed February 2nd, 2024). Either direct orthology to a BindingDB protein based on at least one phylogenomic database or a BLAST hit with E-value < 1 was considered as binding evidence based on BindingDB.

Predictions of ligands corresponding to Pf3D7 AlphaFold (v4) models were taken from the AlphaFill databank^19^, which identifies candidate ligands by searching for sequence homologs in PDB^42^ with known ligands and “transplanting” ligands in regions of local structural homology. AlphaFill excludes common crystallization agents such as polyethylene glycol; in order to focus on AlphaFill hits that are more likely to indicate druggability, we further excluded small ligands with less than ten atoms as well as additional salts, solvents and polymers used for protein crystallization (PDB ligand IDs: 1BO, ACN, ACT, CCN, CIT, CL, DIO, DMS, EOH, FLC, FMT, GBL, HEZ, IPA, JEF, MLA, MLI, MPD, PDO, PEG, PO4, POL, SBT, SIN, SO4, TBU, TLA) listed in McPherson and Gavira 2014^83^. AlphaFill hits having global RMSD < 10 (a measure of structural similarity between the protein of interest and its potential homolog) and local RMSD < 4 (structural similarity of the backbone atoms within 6 Å from the transplanted ligand, after local structural alignment) were considered “confident” hits. Any Pf3D7 protein with at least one confident AlphaFill hit (global RMSD < 10 and local RMSD < 4) to a ligand satisfying the exclusion criteria was classified as having binding evidence based on AlphaFill.

Lastly, inhibitors linked to EC number classes were obtained from BRENDA Enzyme Database^21^ (release 2023.1) by querying EC numbers in the annotated Pf3D7 genes, applicable only to enzymes. Additional ligand types were not considered and for genes with incomplete EC number annotations, all EC numbers matching wildcards were considered. Each Pf3D7 gene with at least one BRENDA EC inhibitor, excluding single-atom ions, was classified as having binding evidence based on BRENDA. Classifications of binding evidence based on orthology or sequence homology to a ligandable protein in BindingDB, presence of confident AlphaFill hit(s), and presence of relevant BRENDA EC inhibitor(s) are listed for each Pf3D7 gene in **Data S1**.

### Identification of human orthologs

Homo sapiens genes (GRCh38, release 39) orthologous to Pf3D7 genes were determined from OrthoMCL, and both sequence and structural similarity were evaluated through pairwise comparison of Pf3D7 and human ortholog AlphaFold (v4) structures in TM-align^45^.

### Definition of hypervariable and core genomic regions

Initial definitions of hypervariable and core regions in the *P.* falciparum 3D7 genome from Miles et al. 2016^84^ were adjusted on a gene-by-gene basis to include most *var*, *rifin*, *stevor*, and *Pfmc-2TM* multigene family members within subtelomeric or internal hypervariable regions. Non-nuclear genome genes were classified according to their respective chromosome (apicoplast or mitochondrial). The genome classifications for each Pf3D7 gene used in this study are listed in **Data S1**.

### Categorization of gene essentiality evidence

For each gene, evidence from each of the three data sources (Zhang et al., PlasmoGEM, and RMgmDB) was classified as either confidently essential, confidently nonessential, unclear, or “no data” if unavailable. Conservative thresholds were used to heuristically categorize genes as confidently essential or nonessential. In the case of the Zhang et al. *piggyBac* insertion mutagenesis dataset, which reports number of transposon insertions in addition to a Mutagenesis Index Score (MIS), genes labelled with the “Non - Mutable in CDS” phenotype were considered confidently essential if 0 insertions were observed, MIS > 0.8, and the phenotype was not noted as “tentative.” Genes labelled as “Mutable in CDS” were considered confidently nonessential if number of insertions ≥ 1, MIS < 0.5, and the phenotype was not noted as “tentative.” Genes measured by the Zhang et al. dataset that did not fulfill either sets of criteria were categorized as having unclear evidence of essentiality. For PlasmoGEM, genes labelled “Insufficient data” were included in the “no data” category. A more complex classification scheme was used to rescue essential genes with the “Slow” phenotype by accounting for relative growth rate. If more than 10% or 20% of the 95% confidence interval for relative growth rate fell below 0.5 for genes labelled “Essential” or “Slow”, respectively, or the PlasmoGEM confidence score < 3 for genes labelled “Essential”, evidence was considered unclear; otherwise, genes with the “Essential” phenotype or “Slow” phenotype with relative growth rate < 0.5 were categorized as confidently essential. Meanwhile, genes labelled “Slow” with relative growth rate ≥ 0.5 were confidently nonessential if the lower bound on relative growth rate > 0.6, genes labelled “Dispensable” were confidently nonessential if either the lower bound on relative growth rate ≥ 0.5 or confidence > 3, and genes labelled “Fast” (suggesting increased growth rate upon disruption) were unilaterally considered nonessential. Finally, for RMgmDB, when the phenotype was not “nt” for “not tested”, evidence was categorized as confidently essential if there was a change in phenotype upon gene modification; if no difference was observed, RMgmDB evidence was considered unclear. Information from the three data sources was integrated to determine gene essentiality bins, which were “full” if all available sources suggest the gene is confidently essential, “anti” if at least one source suggests the gene is confidently non-essential, “partial” if all sources of evidence are unclear, and “no data” if the gene was not tested in any of the three datasets.

### Assessment of gene expression by life cycle stage

To assess gene expression in the asexual blood stage, genes were categorized based on strength of evidence from the Le Roch et al. microarray dataset^41^, which reports expression and logP values for six ABS substages synchronized using two different methods. Previous *P. falciparum* 3D7 gene IDs were mapped to current IDs using PlasmoDB. Genes were considered expressed in ABS if at least one substage showed expression value ≥ 30 and logP ≤ −1, not expressed in ABS if all substages showed expression < 10 or logP > −0.5. Otherwise, evidence was considered unclear; such genes were labelled “potentially expressed” in ABS. The Malaria Cell Atlas Chromium 10x RNA-seq dataset was also incorporated in the web resource and **Data S3**; among the four stages tested (ring, trophozoite, schizont, and gametocyte), genes were considered expressed if median expression > 0, and evidence of expression was considered unclear if the third quartile of RNA-seq expression across cells > 0.

### Candidate target scoring rubric

Ten categories belonging to each data type collected were defined to provide a total of 100 points. Availability of recombinant or in situ protein expression was scored between 0 if no information or 4 if known. Categories for quality of literature and quality of gene essentiality were scored between 0 and 6 points providing higher scores if existent and relevant (for literature) or quantity of evidence (for essentiality). For selectivity, 0 was given if the *Plasmodium* and human ortholog were very similar (though the exact similarity percentage varied between reviewers, a range between 25–83% was observed) and data suggest selectivity could be an issue, or a score of 6 was given if there is a lack of a human ortholog and there was a difference between small molecule inhibitors for *Plasmodium* and human enzyme. Evidence of expression received a score from 3 to 11 if there was evidence in one (ABS), two (ABS and liver stage), or more stages. Target novelty, conservation among species (genetic variation) and assay development ranged from 0–11 depending on amount of prior characterization of the protein, especially as a potential antimalarial target (novelty), or extent of conservation (for genetic variation) or availability of functional assays. Structural information scored from 0–17 depending on availability of crystal structures in any organism, *Plasmodium*, or structure bound to a ligand. Lastly, druggability was scored from no binding pocket known (0) to a maximum of 17 points if a tool compound in *Plasmodium* and growth inhibition was known.

## Data Availability

Data generated and analyzed during the current study are available in the **Supplementary material**. Collected information on protein-coding genes in the Pf3D7 genome are showcased in http://pftargetbrowser.org and can be downloaded from DOI 10.6084/m9.figshare.27190545.v1.
